# Nanomaterials for photocatalysis and applications in environmental remediation and renewable energy

**DOI:** 10.3762/bjnano.14.58

**Published:** 2023-06-13

**Authors:** Viet Van Pham, Wee-Jun Ong

**Affiliations:** 1 HUTECH University, 475A Dien Bien Phu Street, Binh Thanh District, Ho Chi Minh City, Vietnam; 2 Xiamen University Malaysia, Jalan Sunsuria, Bandar Sunsuria, 43900 Sepang, Selangor, Malaysia

Global warming and climate change are increasing global issues. In the last ten years, the intensity of these issues has drawn significant attention from many countries worldwide. One of the factors that cause climate change are industrial processes which need high power to run, and most countries have used fossil fuels for these processes [1]. The use of fossil fuels generates harmful emissions to the environment, such as carbon dioxide (CO_2_), methane (CH_4_), nitrous oxide (N_2_O), nitric oxide and nitrogen dioxide (together termed NO*_x_*), and fluorinated gases (e.g., hydrofluorocarbons, perfluorocarbons, and sulfur hexafluoride) which are currently considered primary sources of environmental [2]. A Global Warming Potential (GWP) measurement was used to compare the global warming effects of different gases. It has been calculated to reflect how long gases remain in the atmosphere, on average, and how strongly it absorbs energy [3]. Besides, the discharge of persistent organic pollutants (POPs) also contributes to water pollution, increasing global environmental pollution. Recently, the reduction and conversion of CO_2_ into fuel as valuable hydrocarbon products has been drawing attention from scientists in materials science, chemical engineering, nanotechnology, and related fields [4]. To reduce contaminants (e.g., air pollution (CO_2_, NO_x_, SO_2_), POPs) there are many routes (e.g., physicochemical approaches, biological fixation, advanced oxidation process, and photocatalysis [5–8]). Among the aforementioned methods, the photocatalysis route is appropriate for treating pollutants, even in atmospheric conditions [9–11]. Moreover, the photocatalysis method is also a potential solution for environmental remediation, carbon emission reduction, and renewable energy production [12–14].

**Figure 1:**
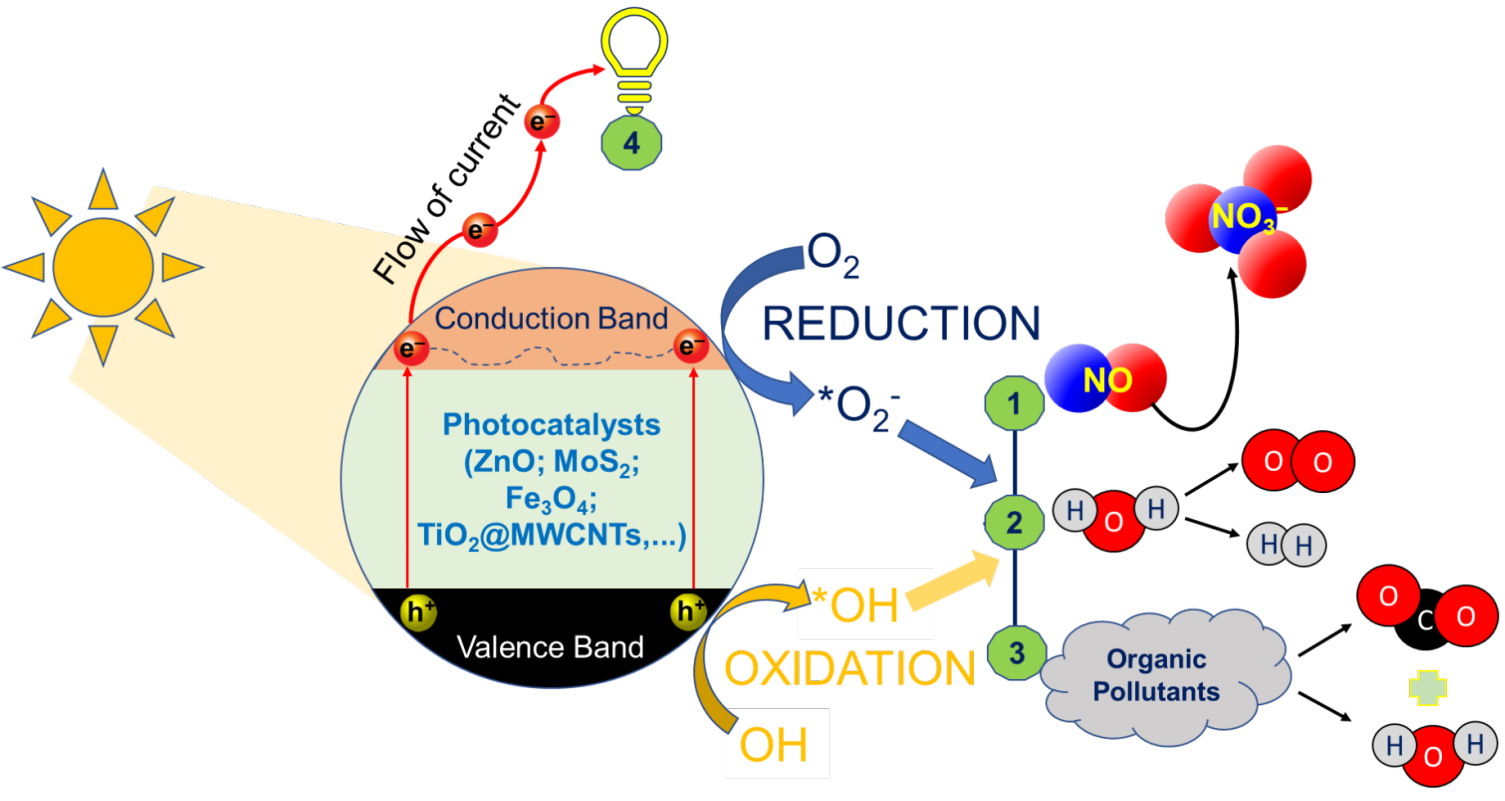
A general photocatalytic mechanism for several possible target processes: (1) NO*_x_* degradation, (2) water splitting for hydrogen and oxygen evolution reactions, (3) degradation of organic pollutants, and (4) solar cell application.

Combining photocatalysts and sunlight irradiation is a potential strategy for water treatment via the effectively infinite energy from the sun and the photocatalysts. Photocatalysis based on nanostructured semiconductors can significantly contribute to tackling several environmental pollution problems, sustainable synthesis, and energy production [2,15–16]. Semiconducting photocatalyst nanomaterials, such as SnO_2_, TiO_2_, MoS_2_, g-C_3_N_4_, and Bi-nanostructures have been proven efficient for a range of applications, including organic pollutant removal, NO*_x_* degradation, renewable energy production, and waste-to-energy conversion [15,17–18]. Figure 1 shows a general photocatalysis mechanism outlining several possible targets (i.e., NO_x_ degradation, water splitting, degradation of organic pollutants, and enhancement of electron generation in a solar-cell application).

This Thematic Issue highlights recent experimental and theoretical developments in using light harvesting by semiconductor materials for sustainable applications; for instance, dye solar cells, solar-driven water splitting, NO*_x_* removal, and contaminant degradation. The synthesis of semiconductor nanomaterials published on this thematic issue indicates a wide range of synthetic routes. The as-prepared nanomaterials with various morphologies demonstrated many preeminent features in the above applications. In detail, the MoS2 with a honeycomb-like structure was first synthesized by an electrochemical route and applied in dye-sensitized solar cells [19], which expressed a higher applicability than that of other studies [20–22]. Besides, Nhu et al. [23] used rosin as a green chemical approach to fabricate ZnO nanoparticles, exhibiting a high photocatalytic activity for both methylene blue (100%) and methyl orange (82.78%) decomposition after 210 min under UV radiation. Moreover, the advantages in the development of advanced materials based on semiconductors (i.e., carbon-modified hexagonal boron nitride (MBN), MgO@g-C3N4, and TiO2@MWCNTs) have indicated a highly efficient photocatalytic performance for phenol removal using a low-power visible LED light source. For NO degradation, a visible light source was used whereas for water splitting natural sunlight was used [24–26]. These results are mentioned as scaling up photocatalytic systems to reach net zero emission goals and the next technology to produce green hydrogen energy [14].

Up-to-date trending topics on photocatalysts based on semiconducting nanomaterials, perovskites, or Bi-based nanomaterials are presented to incentivize fine-tuning of current studies and research works on photocatalytic efficiency of nanomaterials [27]. In addition, this Thematic Issue will undoubtedly provide the reader with novel ideas for developing nanomaterials for environmental remediation and sustainable applications; for instance, dye solar cells, solar-driven water splitting, NO_x_ removal, and contaminant degradation. This Thematic Issue will make a good reference material and be of great use for scientists in nanomaterials fields.

Viet Van Pham and Wee-Jun Ong

Ho Chi Minh City and Sepang, June 2023.
